# A Single E627K Mutation in the PB2 Protein of H9N2 Avian Influenza Virus Increases Virulence by Inducing Higher Glucocorticoids (GCs) Level

**DOI:** 10.1371/journal.pone.0038233

**Published:** 2012-06-13

**Authors:** Jin Tian, Wenbao Qi, Xiaokang Li, Jun He, Peirong Jiao, Changhui Zhang, Guo-Qian Liu, Ming Liao

**Affiliations:** 1 College of Veterinary Medicine, South China Agricultural University, Guangzhou, P. R. China; 2 MOA Key Laboratory for Animal Vaccine Development, Guangzhou, P. R. China; 3 College of Animal Science and Technology, Henan University of Science and Technology, Luoyang, Henan, P. R. China; University of Pittsburgh, United States of America

## Abstract

While repeated infection of humans and enhanced replication and transmission in mice has attracted more attention to it, the pathogenesis of H9N2 virus was less known in mice. PB_2_ residue 627 as the virulent determinant of H5N1 virus is associated with systemic infection and impaired TCR activation, but the impact of this position in H9N2 virus on the host immune response has not been evaluated. In this study, we quantified the cellular immune response to infection in the mouse lung and demonstrate that V_K627_ and rTs_E627K_ infection caused a significant reduction in the numbers of T cells and inflammatory cells (Macrophage, Neutrophils, Dendritic cells) compared to mice infected with rV_K627E_ and Ts_E627_. Further, we discovered (*i*) a high level of thymocyte apoptosis resulted in impaired T cell development, which led to the reduced amount of mature T cells into lung, and (*ii*) the reduced inflammatory cells entering into lung was attributed to the diminished levels in pro-inflammatory cytokines and chemokines. Thereafter, we recognized that higher GCs level in plasma induced by V_K627_ and rTs_E627K_ infection was associated with the increased apoptosis in thymus and the reduced pro-inflammatory cytokines and chemokines levels in lung. These data demonstrated that V_K627_ and rTs_E627K_ infection contributing to higher GCs level would decrease the magnitude of antiviral response in lung, which may be offered as a novel mechanism of enhanced pathogenicity for H9N2 AIV.

## Introduction

H9N2 subtype avian influenza virus was first isolated in turkeys in the U.S. in 1966 [1]. Since 1998, H9N2 viruses have been isolated in pigs and humans in Hong Kong and Mainland China, and the infected displayed an influenza-like illness [2]. These findings indicate the H9N2 avian influenza virus takes on rapid evolution [2]. At the same time, the pressure of vaccine and natural immunity may contribute to substantial virus evolution, which also leads to virus reassorting with other influenza viruses [3]. Multiple studies show some gene segments from newly isolated H9N2 viruses in southeastern and Eastern China possess H5N1 internal genomes [4], [5].

Rapid evolution also leads to enhanced pathogenicity for the virus in mammals and poultry. Evidence shows H9N2 avian-human reassortant virus has enhanced replication and efficient transmission in ferrets [6]. Following adaptation in the ferret, a reassortant virus carrying the surface proteins of an avian H9N2 in a human H3N2 backbone could transmit efficiently via respiratory droplets, creating a clinical infection similar to human influenza infections [7]. In 2010, Hye-Ryoung Kim’s research results showed that three H9N2 reassortant viruses generated from the H5N2 viruses of domestic ducks without pre-adaptation were recovered at high titers in chickens [8].

The evidence on human cases of avian influenza infection in Hong Kong and mainland China leads to more attention to the role of H9N2 avian influenza viruses in human disease [9]. Albeit causing a mild disease in H9N2 virus-infected humans, H9N2 viruses have repeatedly infected humans [10]. Furthermore, some of the H9N2 influenza viruses currently circulating in southern China have molecular features that allow them to preferentially bind to α-2,6-NeuAcGal receptors [11]. Previous studies by Rui Wu et al. indicated that mouse-adapted H9N2 influenza viruses could replicate efficiently and be transmitted among mice through both contact and respiratory droplet routes [12]. Moreover, more evidence shows that H9N2 AIVs causing severe disease in experimentally infected mice without prior adaptation are increasing [13]–[14]. However, the mechanism regarding enhanced pathogenicity to mice for H9N2 virus is less known.

More evidence has demonstrated that PB_2_ residue 627 is a key host range and virulent determinant of influenza A viruses [15] and PB_2_ E627K mutation can directly elevate the enzyme kinetics of influenza polymerase that facilitates virus replication in mammalian cells [16]. In H5N1 virus, a single-amino-acid substitution in PB_2_ residue 627 is associated with systemic infection and impaired T-cell activation in mice [17]. H9N2 AIV prior to adaptation in mice shows multiple amino acid substitutions that include PB_2_ E627K are involved [18]. So far, there are no reports on the impact of a single residue substitution in PB_2_ residue 627 of H9N2 virus on host defense and immune responses. Moreover, H5N1 virus could infect thymus, spleen, and lymphonode and destroy the immune response against virus [19], but H9N2 virus used in our study could not be isolated in these tissues, which suggests the different mechanism of enhanced virulence from H5N1 virus.

Glucocorticoids (GCs) display potent immunomodulatory activities, including the ability to induce T lymphocytes apoptosis and inhibit inflammatory response [20], but high GCs level may be detrimental for host immune response [21]. In our study, we demonstrated that the enhanced virulence for H9N2 AIV correlated with a higher GCs level. Higher GCs titer in plasma of mice induced apoptosis increase in thymus cortex, which impaired the T cells development and led to T cells depletion in lymphoid and lung tissues. Moreover, higher GCs also suppressed the pro-inflammatory cytokines and chemokines level in lungs of mice, which led to the reduction of inflammatory cells infiltration. Finally, the inhibition of host immune defense response contributed to susceptibility to virus infection. GCs were required to protect hosts from lethal immunopathology [21], but the GCs level beyond physiological concentration would destroy the immune response against virus infection, which may be rendered as one of the mechanisms of immunosuppression induced by influenza virus.

**Figure 1:**
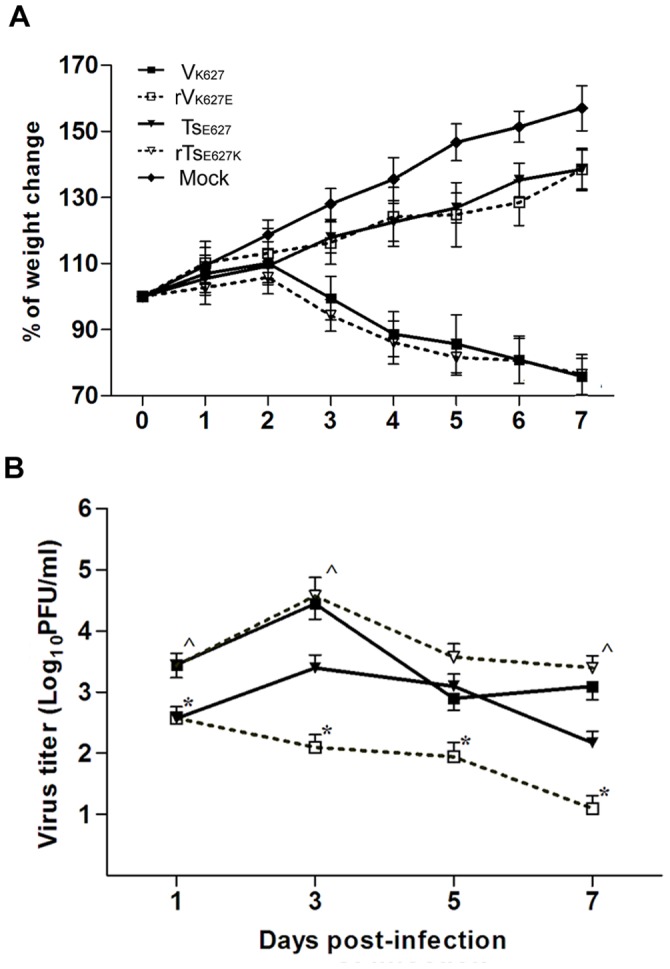
Comparison of weight loss and lung virus titers. Mice (n = 20/group) were inoculated i.n with 10^4^ PFU for V_K627_ (▪), rV_K627E_ (□), Ts_E627_ (▾), and rTs_E627K_ (∇). The weight from eight mice per group was monitored daily (A). Lungs from three mice per group per time point were harvested for virus titration at indicated days p.i. (B). Virus titers were given in units of log_10_PFU per ml. The data shown represents mean ± standard deviation (SD) for three independent experiments. * *p*<0.05 between V_K627_ and rV_K627E_; **^** *p*<0.05 between Ts_E627_ and rTs_E627K_.

## Materials and Methods

### Viruses

The viruses used in study were H9N2 AIV A/chicken/Guangdong/Ts/2004 (Ts_627E_) and A/chicken/Guangdong/V/2008 (V_K627_). Recombinant viruses (rV_K627E_ and rTs_E627K_) were produced by eight-plasmid reverse genetics systems introduced below. Virus stocks propagated in the allantoic cavity of 9- to 11-day-old embryonated specific-pathogen-free hen’s eggs at 37°C. The allantoic fluids were harvested at 48h post-inoculation. The viral titer was determined by plaque assay on MDCK cells (ATCC) in duplicate. In brief, confluent monolayer of MDCK cells were prepared in six-well plates, infected with 10-fold dilutions of virus at 37°C for 2 h. The inoculum was removed, and washed and then overlaid with MEM containing 1% agarose and 2 µg/ml of TPCK-treated trypsin. After plaques had formed at 48–72 h post-infection, the agarose was removed and cells were stained with 0.5% crystal violet in 10% formaldehyde solution. The plaques were visualized and manually counted.

### Construction of Plasmids

A bidirectional transcription vector (pDL) was used to establish eight-plasmid reverse genetic systems. The pDL contains human RNA pol I promoter and murine terminator sequences, which are flanked by the RNA polymerase II promoter of human cytomegalovirus and SV40 late polyadenylation signal. Two BsmB I restriction sites were utilized to clone viral full-length cDNA between RNA pol I promoter and terminator. The viral cDNAs were amplified by RT-PCR with primers containing BsmB I sites (primers are available upon request), and then digested with BsmBI and cloned into the BsmBI sites of the pDL vector. The resulting plasmids (pDL-V-PB2, -PB1, -PA, -HA, -NP, -NA, -M and –NS; pDL-TS-PB2, -PB1, -PA, -HA,-NP, -NA, -M and –NS) were confirmed by sequencing (primers are available upon request). Mutations were introduced into the PB2 gene by site-directed mutagenesis kit (Invitrogen). The resulting plasmids are pDL-V-PB2-627E and pDL-TS-PB2-627K, which were confirmed by sequencing. The plasmids for transfection were prepared by using the Perfectprep Plasmid mini kit (Eppendorf, Hamburg, Germany).

### Generation of Recombinant Viruses

A monolayer of 293T cells (ATCC)with approximately 90% confluence in six-well plates was transfected with 5 µg of the eight plasmids (0.6 µg/each plasmid) by using Lipofectamine 2000 (Invitrogen) according to the manufacturer’s instructions. Briefly, 5 µg of plasmids and 10 µL of lipofectamine 2000 were mixed, incubated at room temperature for 30 min, and then added to the cells. After 6 hours incubation at 37°C, the mixture was replaced with DMEM containing 2% fetal bovine serum and 0.2 µg/mL TPCK-treated trypsin (Sigma-Aldrich). The supernatant was harvested after 2 days incubation and 100 µL of supernatant was injected into an embryonated egg for virus propagation. The inoculated eggs were incubated for 3 days and the allantoic supernatant was collected and tested by hemagglutination assay. The rescued viruses were confirmed by sequencing of the whole viral genome.

### Mice Infections

Four-week-old female BALB/c mice (Experimental Animal Centre of Guangdong Province, P.R. China) were anesthetized with dry ice and intranasally (i.n.) inoculated with 10^4 ^PFU of influenza virus (sublethal inoculum for a prolonged disease course) diluted in 50 µL of sterile, endotoxin-free PBS or 50 µL of sterile PBS (mock group). Mice (n = 8/group) were weighed before infection, and then monitored daily for weight loss as a measure of morbidity. All animal research was conducted under the guidance of CDC’s Institutional Animal Care and Use Committee and in an Association for Assessment and Accreditation of Laboratory Animal Care International- accredited facility. Our animal research in our study had been approved by Guangdong Province Animal Disease Control Center.

### Mifepristone Treatment

The mice (n = 14/group) were treated with 0.1 mg/g (initial weight) of mifepristone (RU486) (Sigma-Aldrich) (suspended in 100 µL of 2% ethyl alcohol) via intraperitoneal administration beginning on day 1 before infection and continued daily until the end of the experiments. The control mice were injected with 100 µL of 2% ethyl alcohol. Both groups were challenged with 10^4 ^PFU of V_K627_ and rTs_E627K_. Weight loss (n = 8/group) was monitored daily as a measure of morbidity. On day 5 post-infection, mice (n = 6/group) from the treatment and control groups were euthanized. The lungs from three mice per group were collected for analysis of virus titer and cytokines. The thymus and lungs from another three mice per group were harvested. Apoptosis and CD4^+^CD8^+^ cells in thymus and T cells and inflammatory in lungs were analyzed by flow cytometry.

**Figure 2:**
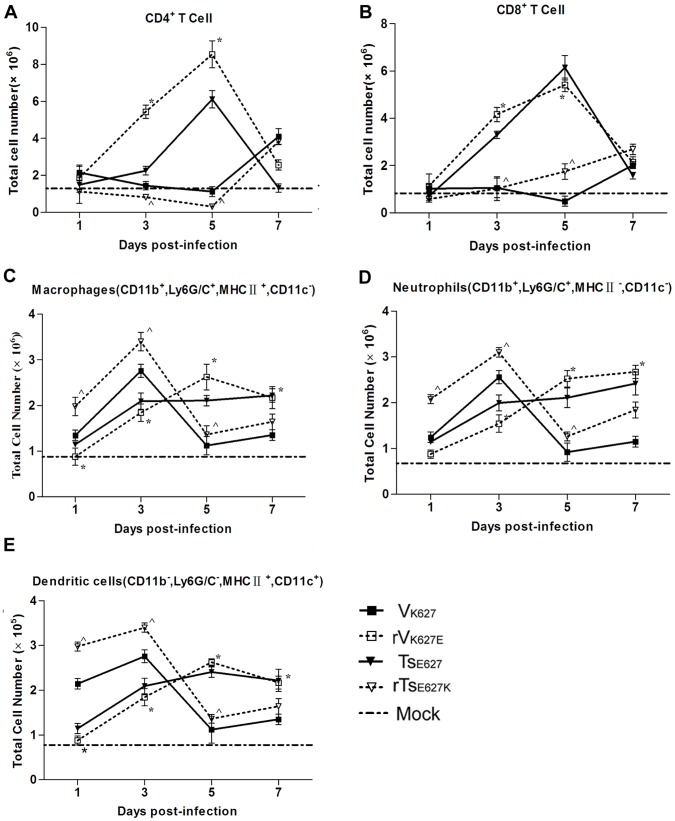
Lung cell characterization following infection. Mice (n = 12/group) were infected i.n. with 10^4^ PFU of V_K627_ (▪), rV_K627E_ (□), Ts_E627_ (▾), and rTs_E627K_ (∇). Lungs from three mice per virus group per time point were harvested and single cell suspensions were prepared. The percentages of T cells and inflammatory cells were determined by appropriate gating on labeled cells. The numbers of CD4^+^ T cells (A), CD8^+^ T cells (B), macrophages (C), neutrophils (D), and dentritic cells (E) were calculated by multiplying the percentage of each cell type by the total number of viable lung cells. Baseline cell numbers from PBS inoculated mice (n = 5) are shown as a dashed lines in each graph. The data shown represents mean ± SD for three independent experiments. * *p*<0.05 between V_K627_ and rV_K627E_; **^** *p*<0.05 between Ts_E627_ and rTs_E627K_.

### Flow Cytometric Analysis

Mice (n = 12/group) were infected with 10^4 ^PFU of V_K627_, rV_K627E,_ Ts_E627_, rTs_E627K_. The peripheral blood, lung and thymus from three mice per group per time point were collected. Lung was washed with cold PBS and homogenized individually in 2 ml of collagenase B (Sigma-Aldrich) at a concentration of 2 mg/ml in RPMI 1640 (Gibco BRL, Grand Island, N.Y.) and incubated for 30 min in a 37°C water bath. Subsequently, the enzyme-digested lung tissues were filtered through a 200-micron nylon mesh to obtain a single cell suspension. The erythrocytes were lysed by treatment with 0.83% of NH_4_Cl-Tris buffer, and the remaining cells were washed and resuspended in PBS. Thymus was gently passed through a 200-micron nylon mesh, lysed with NH_4_Cl-Tris buffer, and single cell suspensions were washed and resuspended in PBS. Next, 0.1 ml of blood or single cell suspensions containing 10^6^ cells was incubated on ice for 10 min with anti-Fc block (anti-CD16/32). Specific cell populations were stained with anti-CD4, anti-CD8 and anti-CD3 for T cells analysis and anti-CD11b, anti-CD11c, anti-Ly6G/6, and anti-MHCII for inflammatory cells analysis. All the mAbs were purchased from eBioscience. After being stained for 30 min at 4°C, the erythrocytes in blood samples were lysed with Optiman C (Beckman), and then the samples were added to 1 ml PBS and analyzed on FACSCalibur flow cytometer (BD Bioscience). Other samples, following staining for 30 min at 4°C, were washed twice, resuspended in 1 ml of 2% paraformaldehyde, and analyzed on FACSCalibur flow cytometer. Inflammatory cells were differentiated by expression of cell-specific markers as indicated in reference [22]. A total of 10,000 gated events were performed in three independent experiments. The number of viable cells per sample was determined by using a Coulter counter (Beckman), and individual cell subsets were calculated by multiplying the percentage of each cell type (as determined by FACS) by the total number of viable cells per tissue.

### Histopathology Analysis

Mice (n = 3/goup) were infected with V_K627_, rV_K627E,_ Ts_E627_, rTs_E627K_ (10^4 ^PFU). At day 5 p.i., three thymuses from each group were fixed in 4% formalin, routinely processed, and embedded in paraffin. Routine hematoxylin-and-eosin-stained sections were examined as previously described [19].

### Analysis of Apoptosis in Thymus

Mice (n = 9/goup) were infected with V_K627_, rV_K627E,_ Ts_E627_, rTs_E627K_ (10^4 ^PFU). At day 3 and 5 p.i., three thymuses from each group were collected. The single cell suspensions from thymus were prepared and 0.1 ml of cell suspensions containing 10^6^ cells was stained with annexin-V and PI (Invitrogen) according to the manufacturer’s instructions. Following staining, the cells were immediately analyzed on a FACSCalibur flow cytometer. The characterization of early apoptotic cells was distinguished as described [23]. A total of 10,000 gated events were performed in three independent experiments. Late apoptotic cells with DNA strand breaks were identified in histological paraffin sections using the in situ terminal deoxynucleotidyl transferase-mediated dUTP-biotin nick end labeling (TUNEL) kit (Sigma-Aldrich). A total of six paraffin sections from three mice per group, sacrificed at day 5 p.i., were prepared according to the manufacturer’s instructions. The brown cells were apoptotic cells.

### Lungs Virus Titrations, Cytokines, and Glucocorticoids Analysis

Mice (n = 12/group) were infected with 10^4 ^PFU of V_K627_, rV_K627E,_ Ts_E627_, rTs_E627K_. The lungs from three mice per group per time point were removed at the indicated time point and stored at −70°C for cytokines and virus titer analysis. At the same time, peripheral blood of mice from the orbital plexus of anesthetized mice was collected and centrifuged to prepare for the plasma. The three lungs from each group per time point were homogenized in 1 ml of cold PBS. The homogenate was pelleted by centrifugation and the virus titer was determined by plaque assay on MDCK cells in duplicate. The titers are reported as plaque forming units per ml PBS (PFU/ml). With the use of ELISA kits (R&D Systems), the clarified lung homogenates were assayed for IL-6, IFN-γ, IL-1β, TNF-α, MIP-1α, MIP-2 and MCP-1 following the manufacturer’s instructions. The levels of cortisol in plasma from three mice per group per time point were measured according to the manufacturer’s instructions by ELISA (Enzo).

### Quantitative Real-time PCR

Mice (n = 12/group) were inoculated i.n. with 10^4 ^PFU of V_K627_ and rTs_E627K_. The three thymuses per group per time point were harvested from day 1 to 7 p.i. Total RNA was isolated from the homogenate using TRIzol reagent (Invitrogen). The allantoic fluids from eggs inoculated with virus and lungs from infected mice were worked as the positive control. The cDNA was achieved with reverse-transcibed kit (Promega). Quantitative real-time PCR was used to determine the expression of beta-actin and influenza virus M gene with SYBR Green PCR kit (TAKARA). The relative expression values of M gene were normalized to the expression value of the β-actin gene. The qPCR programs and primer sequences could be supplied if needed.

### Statistical Analysis

Statistical significance of differences between experimental groups was determined through the use of the paired, nonparametric Student’s t test. *P<*0.05 was thought significant difference.

**Figure 3:**
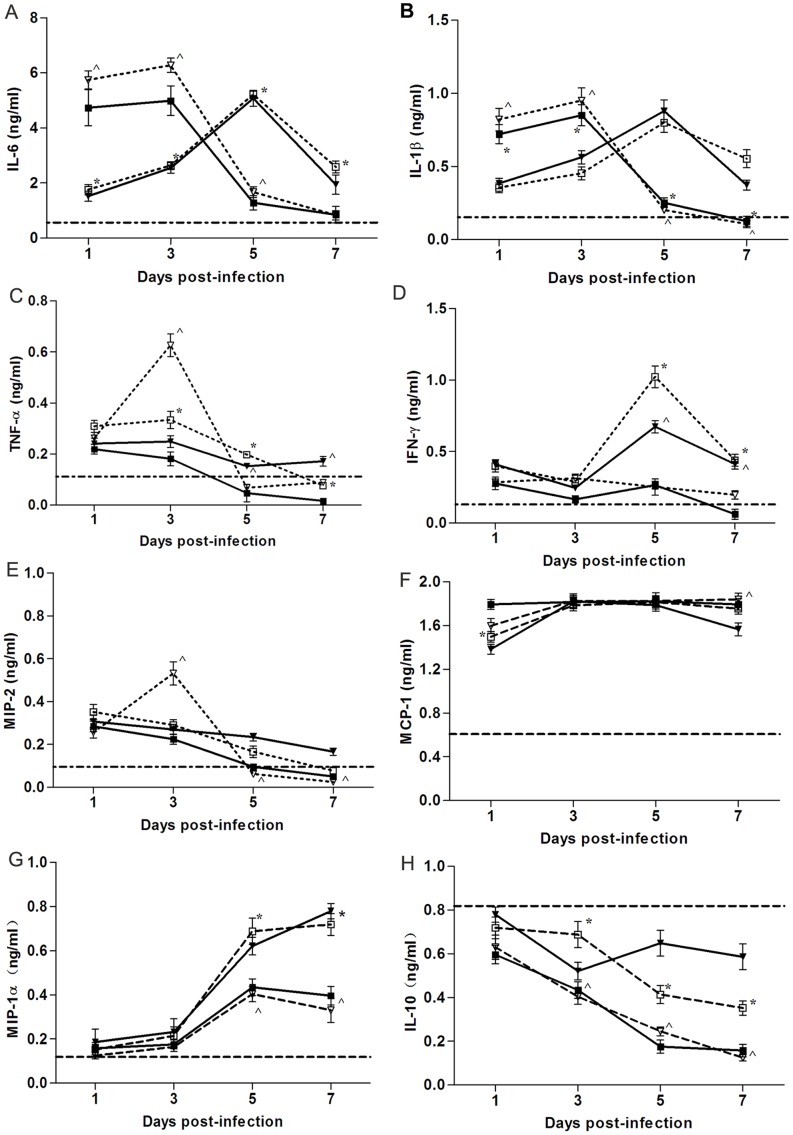
Lung cytokines response. Mice (n = 12/group) were infected i.n. with 10^4^ PFU of V_K627_ (▪), rV_K627E_ (□), Ts_E627_ (▾), and rTs_E627K_ (∇). Lungs from three mice per virus group per time point were harvested and homogenized in 1 ml of PBS. Cytokine levels were measured individually and in duplicate. Baseline cytokine levels from PBS inoculated mice (n = 5) are shown as a dashed line in each cytokine graph. The data shown represents mean ± SD for three independent experiments. * *p*<0.05 between V_K627_ and rV_K627E_; **^** *p*<0.05 between Ts_E627_ and rTs_E627K_.

**Figure 4:**
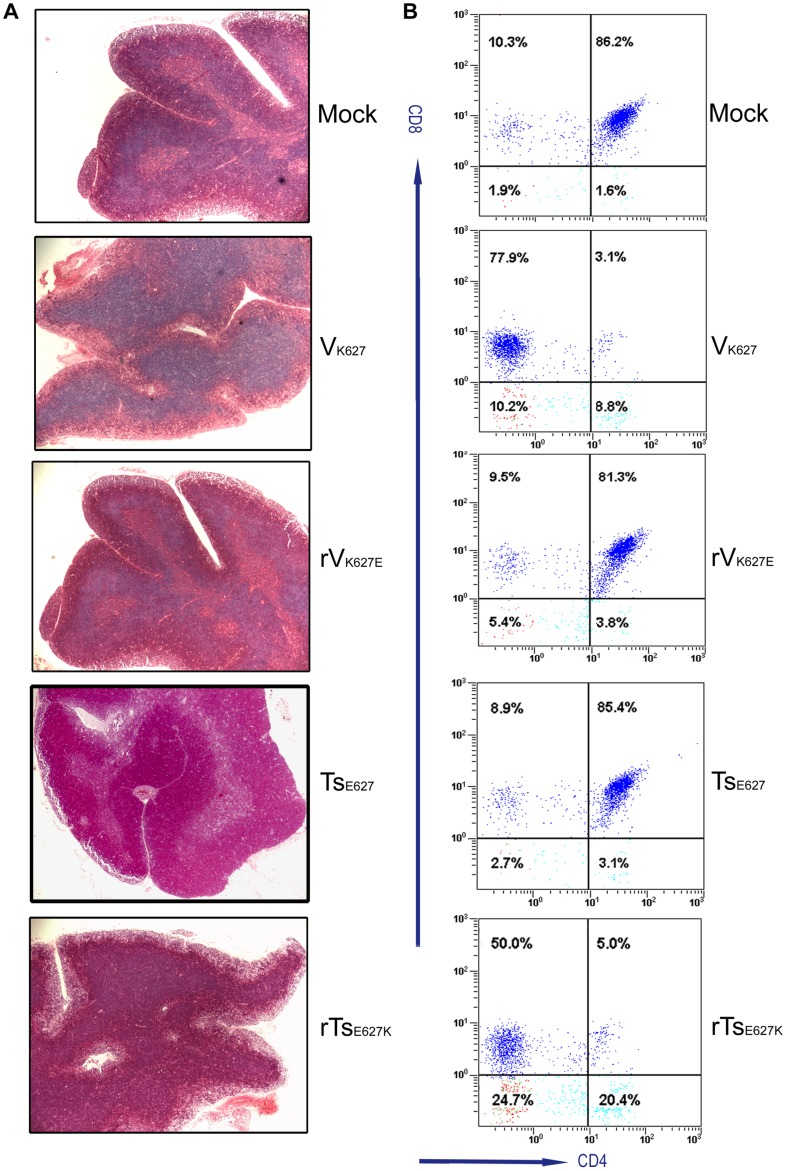
Histopathology and CD4^+^CD8^+^ thymocytes in the thymus. Mice (n = 6/group) were infected i.n. with 10^4^ PFU of V_K627_, rV_K627E_, Ts_E627_, rTs_E627K_, and PBS (mock mice). At 5 day p.i. the thymuses from each group were removed. (A) Three thymuses per group were processed for hematoxylin and eosin. Five sections per tissue were analyzed. Magnification, ×50. (B) Another three thymuses per group were prepared for single cell suspension. Following staining, the percentages of CD4^+^CD8^+^ thymocytes were examined by flow cytometry. Three independent experiments yielded consistent results.

## Results

### A Single-amino-acid Change in the PB2 Protein Affects the Replicative Capacity and Pathogenicity of H9N2 Viruses in Mice

To perform a comparison of mortality of each virus, mice (n = 8/group) were inoculated i.n. with each virus (10^4 ^PFU). The mice infected with V_K627_ or rTs_E627K_ showed the greatest signs of illness, such as ruffled fur and severe morbidity (24.1% and 23.6% weight loss at day 7) (Fig.1A). However, the mice infected with rV_K627E_ or Ts_E627_ showed lighter signs of illness without weight loss (Fig.1A). To determine viral replication in the lungs, lungs from three mice infected with 10^4^ PFU of each virus per group were collected on day 1, 3, 5 and 7 p.i. and viral load in supernatant of lungs homogenizer was quantified in MDCK cells by plaque assay (Fig. 1B). The virus titers in V_K627_-infected lungs were significantly higher (* *p* = 0.031) than titers observed in rV_K627E_-infected lungs over the course of infection. The mutation, E627K in PB_2_, remarkably increased the replicative capacity of rTs_E627K_ in lungs. Compared with that in Ts_E627_-infected mice, lung virus titer in rTs_E627K_-infected mice was increased by approximately tenfold (**^** *p* = 0.029) at day 1 p.i., and the significantly higher virus titers from rTs_E627K_-infected lungs were also observed on days 3 and 7 p.i. All the data showed that the virulence and replicative capacity of the H9N2 virus could be affected by the PB_2_ residue 627.

**Table 1:** Analysis of apoptosis in thymus.

Groups^a^	3 days p.i.^b^		5 days p.i.^b^	
	Annexin V^+^PI^-^±SE (%)	Annexin V^+^PI^+^±SE (%)	Annexin V^+^PI^-^±SE (%)	Annexin V^+^PI^+^±SE (%)
V_K627_	19.59±1.83^c^	1.16±0.12	16.82±1.37 c	2.87±0.32
rV_K627E_	9.43±1.52	1.21±0.17	10.36±1.01	1.84±0.12
Ts_E627_	10.21±1.45^d^	1.12±0.28	11.01±1.13 d	1.13±0.23
rTs_E627K_	20.58±3.03	1.31±0.34	19.19±1.21	2.11±0.07
Mock	11.88±1.84	0.96±0.07	12.11±1.45	0.87±0.06

a Mice (n = 6/group) were i.n. infected with 10**^4 ^**PFU of V_K627_, rV_K627E_, Ts_E627_, rTs_E627K_.

b Mice were euthanized on 3 day p.i. and 5 day p.i., and three thymuses per group per time point were prepared for single cell suspension. Following staining with AnnexinV and PI, apoptotic cells were analyzed by flow cytometry. The early apoptotic cells were determined by annexin V^+^/PI^−^ and late apoptotic cells were determined by annexin V^+^/PI^+^. The data shown represents mean ± SD for three independent experiments.

c *p*<0.05 compared to rV_K627E_;

d *p*<0.05 compared to rTs_E627K_.

### V_K627_ and rTs_E627K_ Infections Decrease the Numbers of T Cells and Inflammatory Cells Infiltrating into Lung

To explore the factors for the enhanced morbidity of H9N2 AIV, the numbers of T cells and inflammatory cells in lungs were quantified (Fig. 2). Following infection with 10^4^ PFU of each virus, lungs from three mice per group were collected at day 1, 3, 5 and 7 p.i. and single cell suspensions were prepared. The percentages of lung immune cells were determined by flow cytometry. After V_K627_ and rTs_E627K_ infection, the numbers of CD4^+^ and CD8^+^ T cells displayed an identical or lower level compared with mock group from day 1 to 5 p.i., but exhibited a progressive increase in rV_K627E_ and Ts_E627_ groups (Figs. 2A and 2B). V_K627_ and rTs_E627K_ infection significantly reduced the numbers of CD4^+^ and CD8^+^ T cells at day 3 p.i. compared with the numbers detected in rV_K627E_ and Ts_E627_ groups. On day 5 p.i., a reduction of at least thrice as few CD4^+^ and CD8^+^ T cells (* *p* = 0.005, **^** *p* = 0.012) was observed in V_K627_ and rTs_E627K_ groups. The inflammatory cells examined in V_K627_ and rTs_E627K_ groups rapidly mounted to the peak from day 1 to 3 p.i., but fell off drastically from day 5 to 7 p.i. (Figs. 2C–2E). In contrast, the numbers in rV_K627E_ and Ts_E627_ groups exhibited a progressive increase (Figs. 2C–2E). These data indicated that V_K627_ and rTs_E627K_ infection resulted in depletion of T cells and inflammatory cells in lung.

**Figure 5:**
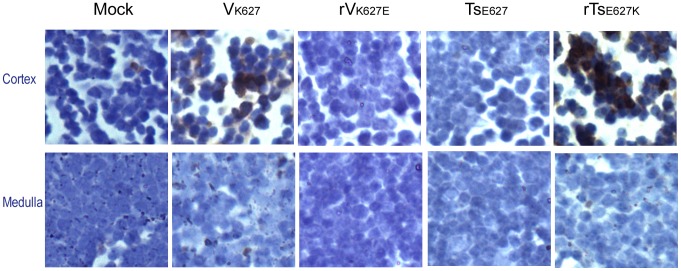
TUNEL assay. Following infection with 10^4^ PFU of V_K627_, rV_K627E_, Ts_E627_, rTs_E627K_, and PBS (mock mice), mice (n = 3/group) were euthanized on day 3 p.i. and thymuses were removed and fixed in 4% formalin. Apoptotic cells were identified in histological sections using the TUNEL assay. Three sections per tissue were analyzed Magnification, ×400. Three independent experiments yielded consistent results.

### V_K627_ and rTs_E627K_ Infections Result in Diminished Proinflammatory Cytokines and Chemokines Production

Many cytokines in the lung are believed to contribute to the recruitment of inflammatory cells. Since the previous experiments established that V_K627_ and rTs_E627K_ infection resulted in a depletion of inflammatory cells, we next determined whether critical cytokine and chemokine responses might also be limited in the lung following infection. After infection with each virus (10^4^ PFU), lungs from three mice per group were collected at day 1, 3, 5 and 7 p.i. and the levels of cytokines (IL-6, IL-1β, TNF-α, IFN-γ) and chemokines (MIP-2, MIP-1α, MCP-1) in lungs were analyzed by ELISA. All cytokines and chemokines were produced well above the constitutive levels 1 day after infection with each virus (Figs. 3A–3G). However, the production of IL-6, IL-1β, IFN-γ and MIP-1α was strikingly reduced in V_K627_ and rTs_E627K_ groups on day 5 to 7 p.i., compared with those in rV_K627E_ and Ts_E627_ groups (Figs. 3A, 3B, 3D, 3G). The levels of TNF-α (Fig. 3C) in V_K627_ group were remarkably lower than the levels in rV_K627E_ group from day 3 to 7 p.i., but the similar decrease was detected only in rTs_E627K_ group at day 5 and 7 p.i. Moreover, the protein levels of MIP-2 were reduced to <25% in the rTs_E627K_ group compared with Ts_ E627_ group at day 5 and 7 p.i. (Fig. 3E). These data revealed V_K627_ and rTs_E627K_ infection led to the diminished production of critical cytokines and chemokines. To examine whether the reduced levels of cytokines and chemokines were caused by elevated IL-10 production, the titer of IL-10 was analyzed (Fig. 3H). Although all the viruses’ infection decreased the production of IL-10, a lower level was observed in V_ K627_ and rTs_E627K_ groups than that in rV_K627E_ and Ts_E627_ groups. So the reduced production of cytokines and chemokines were not caused by higher IL-10 titer.

**Figure 6:**
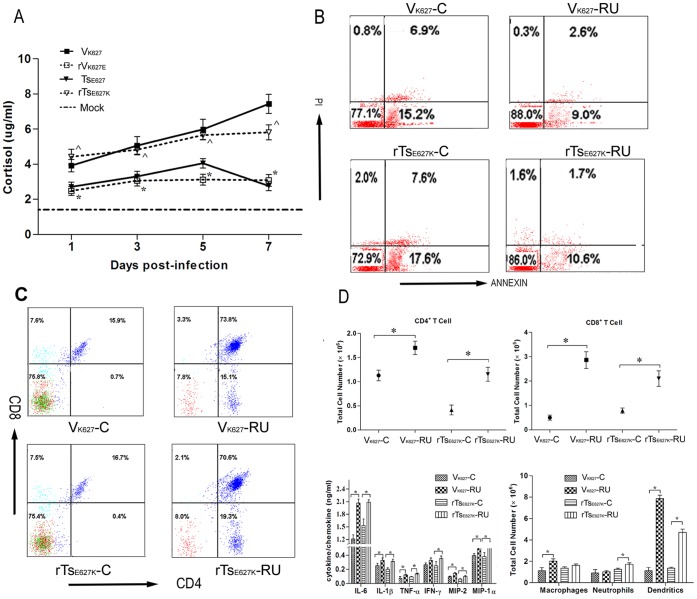
GCs level in plasma and RU486 treatment. (A) Mice (n = 12/group) were infected i.n. with 10^4^ PFU of V_K627_ (▪), rV_K627E_ (□), Ts_E627_ (▾), and rTs_E627K_ (∇). The plasma from three mice per virus group per time point was prepared and GCs level in plasma was measured by ELISA. Baseline GCs levels from PBS inoculated mice (n = 5) are shown as a dashed line in graph. * *p*<0.05 between V_K627_ and rV_K627E_; ^Λ^ *p*<0.05 between Ts_E627_ and rTs_E627K_; (B–D) Following RU486 treatment, mice (n = 6/group) were infected i.n. with 10^4^ PFU of V_K627_ (V_K627_-RU) and rTs_E627K_ (rTs_E627K_-RU). The control groups were named with V_K627_-C and rTs_E627K_-C. All the mice were sacrificed at day 5 p.i. Lungs and thymuses from three mice of each group were removed and single cell suspensions were prepared. The percentages of apoptosis (B) and CD4^+^CD8^+^ thymocytes (C) in thymus and T cells and inflammatory cells in lung were measured by flow cytometry. The numbers of T cells and inflammatory cells (D) were calculated by multiplying the percentage of each cell type by the total number of viable lung cells. Lungs from another three mice in each group were removed and homogenized in 1 ml of PBS. Cytokines levels (D) were measured individually and in duplicate. The data shown in B and C represents three independent experiments and the data shown in A and D represents mean ± SD for three independent experiments. * *p*<0.05 between V_K627_-RU and V_K627_-C; ^*p*<0.05 between rTs_E627K_-RU and rTs_E627K_-C.

**Figure 7:**
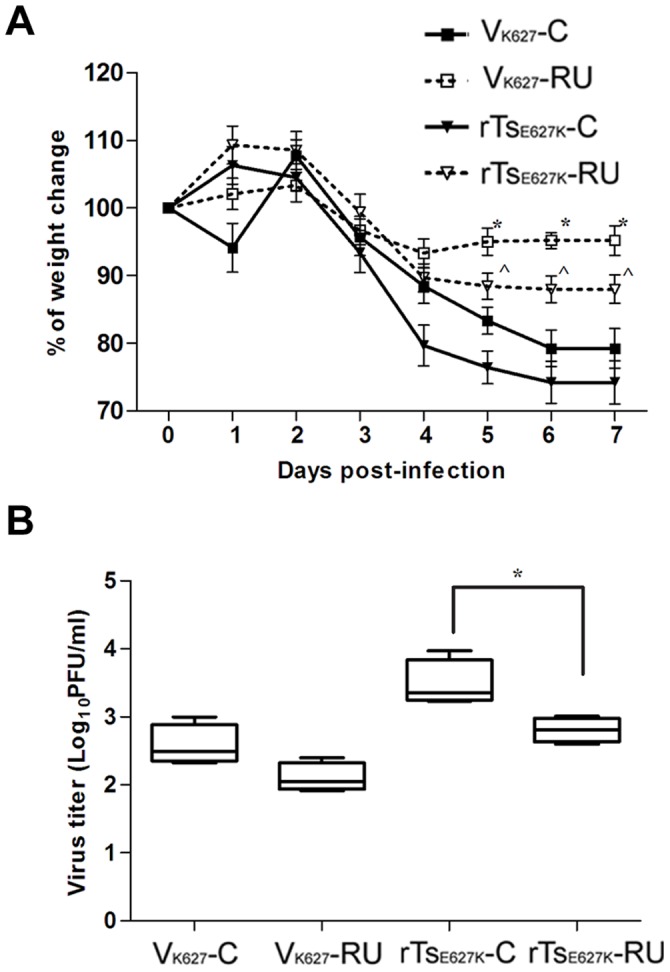
Weight change and viral burden following RU486 treatment. Following RU486 treatment, mice (n = 11/group) were infected i.n. with 10^4^ PFU of V_K627_ (V_K627_-RU) and rTs_E627K_ (rTs_E627K_-RU). (A) Mice (n = 8/group) were weighed daily from 0 day p.i. to 7 day p.i. (B) At 5 day p.i., the lungs from three mice per group were removed and homogenized in 1 ml of PBS, and virus titer was determined by plaque assay. The data shown in A and B represents mean ± SD for three independent experiments. * *p*<0.05 between V_K627_-RU and V_K627_-C; **^** *p*<0.05 between rTs_E627K_-RU and rTs_E627K_-C.

### V_K627_ and rTs_E627K_ Infections Interfere with T Cells Development

The depletion of T cells in V_K627_ and rTs_E627K_ groups were not only observed in lung, but also detected in peripheral blood (Fig. S1A). Whether both virus infections impaired the T cells development in thymus was examined. After infection with each virus (10^4^ PFU), the thymuses from six mice per group were removed at day 5 p.i., and three thymuses were used for analysis of histopathology and another three for analysis of CD4^+^CD8^+^ thymocytes by flow cytometry. Visual atrophy of thymus (Fig. S1B) was observed in V_K627_ and rTs_E627K_ groups. The examination of histopathology (Fig.4A) showed that the normal corticomedullary differentiation was lost following V_K627_ and rTs_E627K_ infection, and the cortex was almost absent. The analysis of lymphocyte populations from thymus showed the percents (4.5±1.5%) of CD4^+^CD8^+^ thymocytes in V_K627_- and rTs_E627K_-infected mice were far lower than the percents (82.3±4.3%) observed in rV_K627E_ and Ts_E627_ infection groups at day 5 p.i. (Fig. 4B). Moreover, the mature CD4^+^ and CD8^+^ T cells in V_K627_ and rTs_E627K_ groups took on remarkably lower numbers than the numbers in rV_K627E_ and Ts_E627_ groups from day 3 to 7 p.i. (Fig. S1C). These data revealed V_K627_ and rTs_E627K_ infection interfered with T cells development and caused T cells lymphopenia in thymus.

### Increased Apoptosis in Cortex of Thymus may be Responsible for the Reduced Percent of CD4^+^CD8^+^ Thymocytes

To check whether the depletion of thymus was induced by increased apoptosis, both flow cytometry and TUNEL assay were performed. After being challenged with each virus (10^4^ PFU), thymuses from three mice per group were collected at day 3 and 5 p.i. and single cell suspensions were prepared. Early apoptosis was analyzed by flow cytometry. The results showed the early apoptosis in V_K627_ and rTs_E627K_ groups had a significant increase compared with that in rV_K627E_ and Ts_E627_ groups at day 3 and 5 p.i. (Table 1). Situ detection of late apoptotic cells in paraffin-embedded thymus sections from two mice per group was achieved by TUNEL assay at day 5 p.i. (Fig. 5). Following infection, many apoptotic cells were pronounced in cortex from V_K627_- and rTs_E627K_-infected mice, but not in medulla. In the rV_K627E_- or Ts_E627_-infected mice, no apoptotic cells could be detected in the cortex and medulla. Cortex of thymus is the location of negative selection for CD4^+^CD8^+^ thymocytes. So, the reduced percent of CD4^+^CD8^+^ thymocytes may result from the increased apoptosis in cortex of thymus.

### The Higher GC Levels in the Plasma of V_K627_- and rTs_E627K_- Infected Mice are an Important Factor for the Increased Apoptosis in Thymus and the Decreased Infiltration of T Cells and Inflammatory Cells in Lung

Apoptosis is regarded as a host defense mechanism against virus infections that works by removing foreign nucleic acids from an infected host [24]. Following infection with 10^4 ^PFU of V_K627_ and rTs_E627K_, the levels of virus nucleic acids in thymuses from three mice per group were analyzed from day 1 to 7 p.i. by qPCR. But the virus nucleic acids could not be detected during the whole course of infection (data not shown).

We subsequently found the mice infected with each virus showed a sustained increase level of GCs in the plasma compared with the mock group (Fig. 6A). But the levels of GCs in V_K627_ and rTs_E627K_ groups were significantly higher than rV_K627E_ and Ts_E627_ groups from day 1 to 7 p.i. So we hypothesized the increased apoptosis in thymus was caused by higher GCs levels. Next, whether blocking the glucocorticoid receptors (GR) could decrease thymocytes apoptosis after V_K627_ or rTs_E627K_ infection was examined. The RU48-treated mice were infected i.n. with 10^4 ^PFU of V_K627_ (V_K627_-RU) and rTs_E627K_ (rTs_E627K_-RU). At day 5 p.i., the lungs and thymuses from three mice per group were harvested and single cell suspensions were prepared. The early and late apoptosis in thymus of V_K627_-RU or rTs_E627K_-RU groups was strongly decreased at day 5 p.i. compared with mock groups (Fig. 6B). Importantly, the percentages of CD4^+^CD8^+^ thymocytes in both RU486-treated groups recovered to 75.2±5.5%, which was significantly higher than mock groups (11.5±2.1%) (Fig. 6C). RU486 treatment also significantly increased the infiltration of T cells in the lungs (Fig. 6D). Endogenous and pharmacologic GCs limit inflammatory cascades by modulating a wide range of inflammatory molecules, including many cytokines [21]. Indeed, blockade of GR resulted in the dramatic increase in production of inflammatory cytokines (IL-6, IL-1β, TNF-α) and chemokines (MIP-1α, MIP-2) (Fig. 6D). Moreover, the dentritic cells in RU486-treated groups had a remarkable increase (>3 folds) (* *p* = 0.0002, **^** *p* = 0.012). But the significant increase of macrophage was observed only in V_K627_-RU group, and the similar increase of neutrophils was only observed in rTs_E627K_-RU group (Fig. 6D). So, the higher level of GCs played an important role in the decreased T cells, and inflammatory cells in V_K627_- and rTs_E627K_-infected lungs.

### Blockade of Glucocorticoid Receptors Results in Protection to V_K627_ and rTs_E627K_ Challenge in Mice

To examine the effect of blockade of GR on virus infection, RU486-treated mice (n = 11/group) were challenged with 10^4 ^PFU of V_K627_ and rTs_E627K_. The weight loss (n = 8/group) was monitored daily. At day 5 p.i., virus loads of lungs from three mice per group were determined by plaque assay. We noted that the V_K627_-RU- and rTs_E627K_-RU-infected mice experienced less weight loss compared with mock mice from day 4 to 7 p.i. (Fig. 7A). The virus loads of lungs in both RU486-treated groups were decreased at day 5 p.i. but significant differences (^*p* = 0.013) were observed only between rTs_E627K_-RU and rTs_E627K_-C (Fig. 7B).

## Discussion

The PB_2_ residue 627 has been identified as an important determinant of host range restriction [25] and virulence and replicative efficiency in animal models [15], [17], [26]. In the in vivo infection experiment, the replicative efficiency and virulence of rV_K627E_ was obviously lower than V_627K_ and the replicative efficiency and virulence of rTs_E627K_ was significantly enhanced compared with Ts_E627_, which suggested that PB_2_ residue 627 substitutions affected the replicative efficiency and virulence in vivo. However, it is more important to understand how the presence of lysine at position 627 of the PB2 protein affects virus-host interactions and is sufficient to allow the virus to replicate quickly. In our study, we found PB_2_ residue 627 substitution affected host immune defense response and contributed to susceptibility to virus infection. However, whether there were other amino acids in the genomes of H9N2 AIV that could contribute to same effect needs to be investigated further.

The proinflammatory cytokine response is responsible for recruiting immune effector cells to clear the virus infection [27]. Nevertheless, this response, with inappropriate activation or inefficient regulation, may contribute to severe lung viral pneumonia and serious complications of infection [28], [29]. It has been proposed that the increased pathogenicity for 1918 and H5N1 influenza virus infection is related to excessive early cytokine response, immune cell recruitment, and poor outcome [30]. But highly lethal H5N1 influenza (HK483) could reduce inflammatory cells recruitment, which is considered as the cause of virus not being cleared from tissues [19]. So, the enhanced morbidity for V_K627_ and rTs_E627K_ infections was associated with the reduction of inflammatory cells in lungs.

Recently several studies have established a role for CD8^+^ T cells during the innate immune response against bacterial and parasite infection [31]. A lack of CD8^+^ T cells led to increased viral replication and morbidity in mice infected with A/Puerto Rico/8/34 (PR8). Moreover, CD4^+^ T cells can direct CD8^+^ T cells response by secreting TypeI panel cytokines (IFN-γ, IL-2, TNF-α), modulating the magnitude and duration of CD8^+^ T cells response and driving B cells production of antibody to neutralize viral particles [32]. The insufficient infiltration of CD4^+^ and CD8^+^ T cells in lungs may impair the host immune response. So, suppressive T cells response also contributed to the enhanced morbidity for V_K627_ and rTs_E627K_ infections.

Macrophages and neutrophils can secrete chemokines and cytokines that can act in an autocrine fashion, which in turn can promote the migration of those cells and other leukocytes into lung tissue [28]. Contrary to the widely recognized theory, the expression of cytokines is mainly regulated by pulmonary endothelium [33]. We found the levels of proinflammatory cytokines and chemokines examined in the lung were significantly inhibited at day 5 and 7 p.i. So, fewer cells migrating into lung may be associated with the suppressive expression of cytokines in lung of V_K627_ or rTs_E627K_-infected mice. The mice deficient in either IL-1β, TNF-α, IFN-γ increased their mortality due to influenza virus infection compared with wild-type control mice [34]. The reduced level for IL-6, IL-1β, TNF-α, and IFN-γ in lung may be a reason for the enhanced morbidity in V_K627_ and rTs_E627K_ infections.

Leukopenia has been demonstrated following infection with many of viruses, and a transient leukopenia could occur following infection with human influenza subtypes in humans [19], [35]. However, the mechanism of lymphopenia remains less known. To investigate a possible mechanism for T cells lymphopenia in peripheral and lymphoid tissue after V_K627_ and rTs_E627K_ infection, we focused on the thymus. In addition to the thymus suffering from severe atrophy, the histopathology examination of thymus revealed that V_K627_ or rTs_E627K_ infection induced the cortex of thymus to be reduced. More importantly, the percent of CD4^+^CD8^+^ thymocytes in V_K627_- or rTs_E627K_-infected mice underwent a processive reduction. So, inability of the thymus to reproduce the peripheral T lymphocyte compartment may represent one mechanism of T cells reduction in peripheral tissues [35].

Influenza virus-induced apoptosis was primarily thought to be a host defense mechanism to limit virus replication and clear viruses from the host [36]; however, the virus has abilities not only to overcome but to utilize apoptosis for its efficient replication [37]. Because H5N1 virus could be detected in these lymphoid organs, it is difficult to judge increased apoptosis induced by virus directly or indirectly [19]. In our research, the data demonstrated that V_627K_ and rTs_627K_ did not directly participate in increasing apoptosis in thymus because no virus nucleic acids were detected in thymus. GCs are important for T cell selection development and AICD [38], [39]. GCs have long been known to induce cell death (apoptosis) in the thymus (40). The CD4^+^CD8^+^TCR^low^ subset, although expressing less than half the GR density of CD4^–^CD8^–^TCR^–^ cells, is the most sensitive subset to GCs-induced apoptosis [41], with which the results in our study were consistent. Moreover, after RU486 treatment, the apoptosis in the thymus of mice infected with V_K627_ or rTs_E627K_ was decreased, and the CD4^+^CD8^+^ thymocytes and lung T cells were increased at day 5 p.i. So the GCs play an important role in increased apoptosis indirectly induced by influenza virus infection.

In the infection with herpes simplex virus-1 (HSV-1), the exposure to stress or corticosterone in the earliest stages of infection is sufficient to suppress the subsequent antiviral immune response in a glucocorticoid receptor-dependent manner [42]. The ability of popliteal lymph nodes-derived dendritic cells to prime HSV-1–specific CD8+ T cells is functionally impaired and the administration of the GR antagonist completely prevented stress from reducing the numbers of activated, functional CD8+ T cells [42]. Recent study proves early GCs exposure would increase the risk of developing critical disease in humans infected by pandemic influenza A (H1N1) virus infection [43]. Influenza virus infection could trigger a stress response leading to a sustained increase in serum GCs, which compromised innate immune response, but virus-induced GCs production is necessary to prevent lethal immunopathology during coinfection [21]. So, we hypothesized the physiological level of GCs may be beneficial to regulating host immune response by modulating the production of cytokines, but higher levels may be detrimental to host defense against virus infection. After RU486 treatment, the titers of cytokines were increased, which resulted in enhancement of inflammatory cells infiltration. More importantly, the virus load in lungs had a reduced trend and the weight loss of mice was decreased following treatment with RU486. But the two cell types other than dentritic cells did not fully recover the same level as in rV_K627E_- or Ts_E627_-infected mice. So, the higher GCs level triggered by the V_K627_ or rTs_E627K_ infection may not be the sole factor causing increased virulence for H9N2 virus expressing PB_2_ 627K, but it played an important role in enhancing pathogenicity in mice.

The interaction between the neuroendocrine and immune systems is now well demonstrated. More evidence has placed hormones and neuropeptides among potent immunomodulators, participating in various aspects of immune system function in both health and disease [44]. Hypothalamic- pituitary-adrenal (HPA) axis can be stimulated following infection of many viruses, resulting in the release of adrenal GCs [45]. The production of IL-6 and IL-1β after virus infection can stimulate HPA axis, which leads to the release of GCs [46]. However, another study revealed that the high level of serum GCs induced by influenza virus infection was in part independent of systemic inflammatory cytokines [21]. So, the exact mechanism linking infection-induced tissue damage to the HPA axis activation is currently unknown.

## Supporting Information

Figure S1 **Analysis of T cells in blood and thymus of infected mice.** Mice (n = 12/group) were infected i.n. with 10^4^ PFU of V_K627_ (▪), rV_K627E_ (□), Ts_E627_ (▾), and rTs_E627K_ (∇). Blood and thymuses from three mice per group per time point were collected. The percents of CD4^+^ and CD8^+^ T cells in blood (A) were analyzed by flow cytometry. The morphology of thymuses (B) was photoed and single cell suspension was prepared. The percentages of T cells (as determined by appropriate gating on labeled cells) were examined and the numbers of T cells (C) in thymus were calculated by multiplying the percentage of each cell type by the total number of viable thymus cells. Baseline from PBS inoculated mice is shown as a dashed line in each graph. The data shown in A and B represents mean ± SD for three independent experiments. * *p*<0.05 between V_K627_ and rV_K627E_; **^** *p*<0.05 between Ts_E627_ and rTs_E627K_.
(TIF)
