# Supplementary figures and images for: A Single E627K Mutation in the PB2 Protein of H9N2 Avian Influenza Virus Increases Virulence by Inducing Higher Glucocorticoids (GCs) Level
Source: PLoS One. 2012 Jun 13;7(6):e38233. doi: 10.1371/journal.pone.0038233 (PMC3374829; doi:10.1371/journal.pone.0038233)

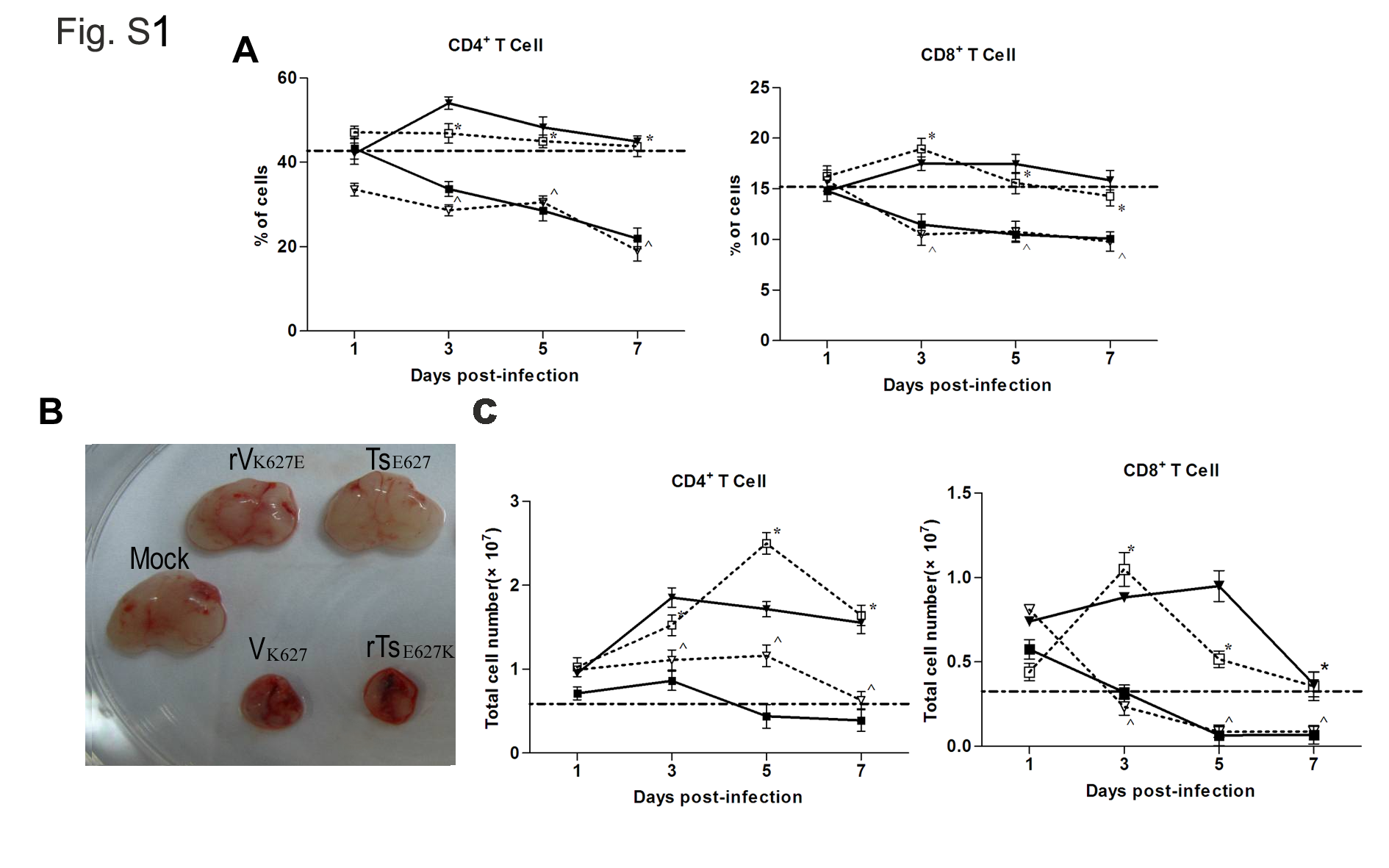

Supplement: Figure S1 — Analysis of T cells in blood and thymus of infected mice. Mice (n = 12/group) were infected i.n. with 104 PFU of VK627 (▪), rVK627E (□), TsE627 (▾), and rTsE627K (∇). Blood and thymuses from three mice per group per time point were collected. The percents of CD4+ and CD8+ T cells in blood (A) were analyzed by flow cytometry. The morphology of thymuses (B) was photoed and single cell suspension was prepared. The percentages of T cells (as determined by appropriate gating on labeled cells) were examined and the numbers of T cells (C) in thymus were calculated by multiplying the percentage of each cell type by the total number of viable thymus cells. Baseline from PBS inoculated mice is shown as a dashed line in each graph. The data shown in A and B represents mean ± SD for three independent experiments. * p<0.05 between VK627 and rVK627E; ^ p<0.05 between TsE627 and rTsE627K. (TIF) [file pone.0038233.s001.tif]
